# Supplementary material for: Online games: a novel approach to explore how partial information influences human random searches
Source: Sci Rep. 2017 Jan 6;7:40029. doi: 10.1038/srep40029 (PMC5216393; doi:10.1038/srep40029)
Supplement: Supplementary Information [file srep40029-s1.pdf]

Supplementary information for "Online games: a novel approach to explore how partial information influences random search processes"

Ricardo Martínez-García, Justin M. Calabrese, and Cristóbal López

N_y	Number of rounds
0	58
3	81
5	65
7	68
9	79
11	77
13	72

Supplementary Table I: Square neighborhoods. Distribution of the 500 rounds with depending on the size of the yellow regions

N_y	Number of rounds
6	95
17	65
34	64
56	40
70	37

Supplementary Table II: Randomized neighborhoods. Distribution of the 301 rounds depending on the size of the yellow regions
